# Neutralisation of the SARS-CoV-2 Delta variant sub-lineages AY.4.2 and B.1.617.2 with the mutation E484K by Comirnaty (BNT162b2 mRNA) vaccine-elicited sera, Denmark, 1 to 26 November 2021

**DOI:** 10.2807/1560-7917.ES.2021.26.49.2101059

**Published:** 2021-12-09

**Authors:** Ria Lassaunière, Charlotta Polacek, Jannik Fonager, Marc Bennedbæk, Lasse Boding, Morten Rasmussen, Anders Fomsgaard

**Affiliations:** 1 Virus and Microbiological Special Diagnostics, Statens Serum Institut, Copenhagen, Denmark; 2 Danish National Biobank, Statens Serum Institut, Copenhagen, Denmark

**Keywords:** SARS-CoV-2, COVID-19, Delta, variant, AY.4.2, coronavirus, virus neutralization, E484K mutation

## Abstract

Several factors may account for the recent increased spread of the SARS-CoV-2 Delta sub-lineage AY.4.2 in the United Kingdom, Romania, Poland, and Denmark. We evaluated the sensitivity of AY.4.2 to neutralisation by sera from 30 Comirnaty (BNT162b2 mRNA) vaccine recipients in Denmark in November 2021. AY.4.2 neutralisation was comparable to other circulating Delta lineages or sub-lineages. Conversely, the less prevalent B.1.617.2 with E484K showed a significant more than 4-fold reduction in neutralisation that warrants surveillance of strains with the acquired E484K mutation.

The severe acute respiratory syndrome coronavirus 2 (SARS-CoV-2) Delta variant of concern (VOC) (Phylogenetic Assignment of Named Global Outbreak Lineages (Pangolin) designation B.1.617) sub-lineage AY.4.2 recently accounted for an increased proportion of coronavirus disease (COVID-19) cases caused by the Delta variant in the United Kingdom (UK), increasing from 3.8% to 20.3% in the weeks of 19 September 2021 and 15 November 2021, respectively [1,2]. The Delta sub-lineage AY.4.2 has spread globally, evidenced by over 48,000 sequences uploaded onto GISAID from 42 countries [3] and frequencies above 1% observed in Romania and Poland [4] as at 23 November 2021. A Delta strain bearing a known neutralisation-resistant mutation, E484K [5,6], has also established small clusters in the UK [7] and contributed to breakthrough infections in Italy [8]. Here, we investigate the sensitivity of the Delta sub-lineage AY.4.2 and Delta lineage B.1.617 with the E484K mutation (+ E484K) to neutralisation by SARS-CoV-2 Comirnaty (BNT162b2 mRNA, BioNTech-Pfizer, Mainz, Germany/New York, United States (US)) vaccine-induced anti-sera.

## Cohort of vaccinated Danish residents

SARS-CoV-2 virus neutralisation capacity was assessed using anti-sera from Danish residents vaccinated with Comirnaty, the predominant SARS-CoV-2 vaccine used in Denmark (85%). The cohort (n = 30), vaccinated with two doses between 18 January and 15 May 2021, was selected to represent a broad age range (20–91 years; median: 42 years; interquartile range (IQR): 35–63; 7/30 donors were men) and varied neutralisation reactivity against an early pandemic strain that is highly homologous to the vaccine strain (D614G; Table) [9]. The time between the second vaccination and sampling ranged from 30 to 100 days (median: 61 days; IQR: 51–64). All donors developed anti-SARS-CoV-2 spike antibodies after vaccination according to the Wantai Total Ab ELISA assay (Beijing Wantai Biological Pharmacy, Beijing, China) using the manufacturer’s recommended cut-off for positivity [10,11]. The samples were excess material from diagnostic testing conducted at Statens Serum Institut, Denmark. Samples were taken for diagnostic purposes unrelated to COVID-19; however, they are linked to the Danish Vaccination Registry, which enables surveillance of immune escape by vaccine-induced antibody responses.

**Table:** SARS-CoV-2 viral isolates evaluated for neutralisation using Comirnaty (BNT162b2 mRNA) vaccine sera, Denmark, 1–26 November 2021

Variant	Pango lineage designation	Strain
Early pandemic strain (D614G)	B.1	SARS-CoV-2/Hu/Denmark/SSI-H1
Alpha	B.1.1.7	SARS-CoV-2/Hu/Denmark/SSI-H14
Beta	B.1.351	hCoV-19/Netherlands/NoordHolland_10159/2021
Gamma	P.1	SARS-CoV-2/Hu/Denmark/SSI-H26
Delta	B.1.617.2	SARS-CoV-2/Hu/Denmark/SSI-H11
Delta	AY.4	SARS-CoV-2/Hu/Denmark/SSI-H38
Delta	AY.4.2	SARS-CoV-2/Hu/Denmark/SSI-H37
Delta	B.1.617.2 + E484K	SARS-CoV-2/Hu/Denmark/SSI-H41

Pango: Phylogenetic Assignment of Named Global Outbreak Lineages (Pangolin) designation; SARS-CoV-2: severe acute respiratory syndrome coronavirus 2.

## Neutralisation assay

Virus neutralisation was tested against SARS-CoV-2 viruses isolated on Vero E6 cells (Table). All virus stocks were sequenced to confirm the presence of lineage-specific mutations and the absence of cell culture-derived mutations. Two-fold serially diluted serum samples (range: 1:10 to 1:1,280) were incubated with 300 x 50% tissue culture infectious dose (TCID50) for 1 h and added to a monolayer of Vero E6 cells in 96-well tissue culture plates. After 24 h, virus inhibition was measured in a validated in-house anti-SARS-CoV-2 nucleocapsid protein ELISA, following the principle of the World Health Organization influenza microneutralisation protocol [12]. This SARS-CoV-2 microneutralisation assay has a comparable performance relative to other neutralisation assays used in European laboratories (Laboratory 4 in ref [13]). The ELISA signal was unaffected by amino acid substitutions present in the nucleocapsid protein of some SARS-CoV-2 isolates. Exact 50% neutralisation titres were calculated using four-parameter logistic regression and titres below the lower limit of quantitation (1:10) were assigned a titre of 1:5. Titres were compared using the nonparametric Friedman test for paired measurements followed by Dunn’s multiple comparison test. Adjusted p values are reported.

## AY.4.2 virus neutralisation relative to other Delta variants

**Figure 1:**
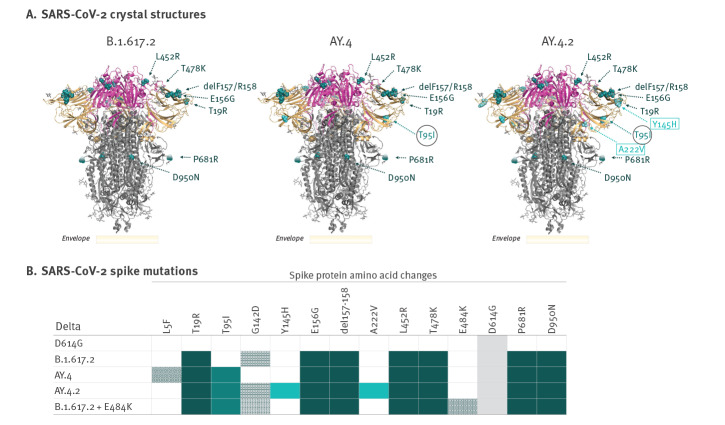
Relative positions of spike mutations of SARS-CoV-2 Delta lineages B.1.617.2, AY.4 and AY.4.2, Denmark, 2021

**Figure 2:**
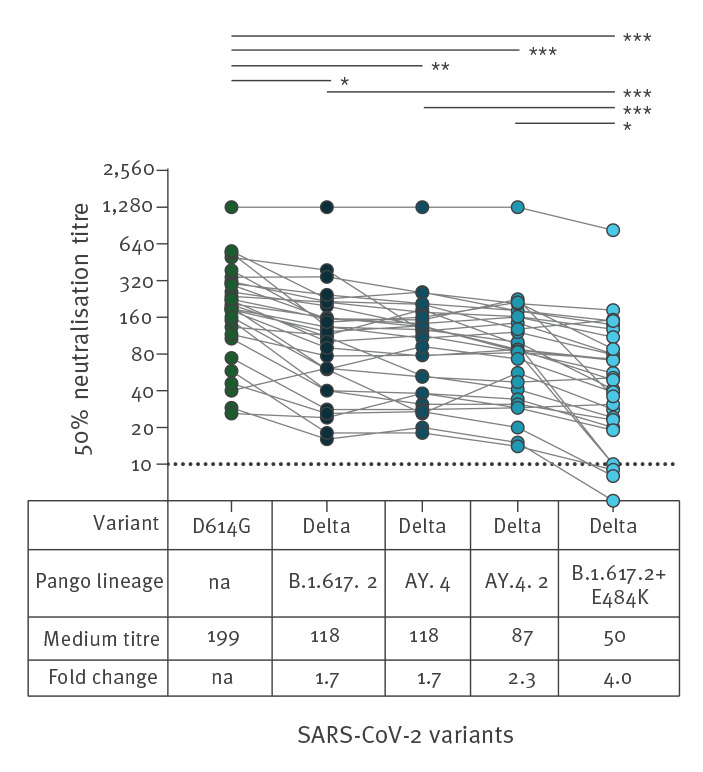
SARS-CoV-2 neutralising antibody titres against Delta sub-lineage AY.4.2 after the second dose of Comirnaty (BNT162b2 mRNA) vaccine, Denmark, 1–26 November 2021 (n = 30)

The Delta sub-lineage AY.4.2 bears the same spike mutations as the Delta lineage AY.4 with the addition of Y145H and A222V in the N-terminal domain (Figure 1). The A222V mutation occurred in SARS-CoV-2 variants that emerged in June 2020, but has been shown not to contribute to increased transmissibility or immune escape in those variants [14]. A functional consequence for Y145H remains to be established. Relative to the early pandemic strain (D614G), the AY.4.2 virus had a 2.3-fold reduction in median neutralisation titres (median titre: 199 vs 87; p < 0.001) (Figure 2). The titres for AY.4.2 did not differ significantly from those measured for the parental Delta lineage B.1.617.2 (median titre: 87 vs 118; p > 0.050) or Delta sub-lineage AY.4 (median titre: 87 vs 118; p > 0.050). We further evaluated a B.1.617.2 lineage isolate that contains the E484K amino acid substitution in the receptor-binding domain. In contrast to AY.4.2, the B.1.617.2 strain with E484K had a significant reduction in virus neutralisation titres relative to D614G (4.0-fold) and all other Delta strains tested – B.1.617.2 (2.3-fold), AY.4 (2.3-fold), and AY.4.2 (1.7-fold) (p < 0.050 for all comparisons).

SARS-CoV-2: severe acute respiratory syndrome coronavirus 2.

A. Pre-fusion SARS-CoV-2 Delta (Pango lineage designation B.1.617.2) spike crystal structures with receptor binding domain (magenta), N-terminal domain (beige), S2 domain (grey), and amino acid substitutions and deletions relative to the Wuhan-Hu-1 reference sequence (indicated by cyan spheres). Sources: Spike protein mutations are defined at Outbreak.info [4], which lists B.1.617.2 VOC-specific spike mutations, and AY.4.2 mutations are defined by the United Kingdom Health Security Agency [1]. Dark turquoise indicates B.1.617.2 lineage mutations, light turquoise (in black circles) indicates AY.4 sub-lineage mutations, and light cyan (in boxes) indicates AY.4.2 sub-lineage mutations.

B. Spike mutations of SARS-CoV-2 Delta variant clinical isolates sequenced in Denmark between 13 March 2020 (D614G) and 12 October 2021, which were used to evaluate virus neutralisation of an early pandemic strain D614G, and Delta variants B.1.617.2, AY.4, AY.4.2, and B.1.617.2 + E484K. Dark turquoise boxes indicate Delta lineage-defining spike mutations present in all Delta lineages/sub-lineages evaluated, light turquoise boxes indicate the AY.4 sub-lineage mutation, light cyan boxes indicate the AY.4.2 sub-lineage mutations, patterned boxes indicates other mutations present in the strains evaluated, and grey boxes indicates the D614G mutation that predominated globally since April 2020.

na: not applicable; Pango: Phylogenetic Assignment of Named Global Outbreak Lineages (Pangolin) designation; SARS-CoV-2: severe acute respiratory syndrome coronavirus 2.

Virus neutralisation for each serum sample was tested against clinical isolates representing an early pandemic strain (D614G), the parental Delta lineage (Pango lineage designation B.1.617.2), Delta lineage AY.4, Delta sub-lineage AY.4.2, and a Delta lineage B.1.617.2 bearing the neutralisation-resistant spike mutation E484K. Light grey lines connect data from each sample for the different virus isolates. Fold changes are relative to D614G. Serum samples were each taken from a different individual (n = 30) and tested in a two-fold serial dilution ranging from 1:10–1:1,280. The dotted line represents limit of detection (< 1:10). *p < 0.05, **p < 0.01, ***p < 0.001.

## AY.4.2 virus neutralisation relative to variants of concern

**Figure 3:**
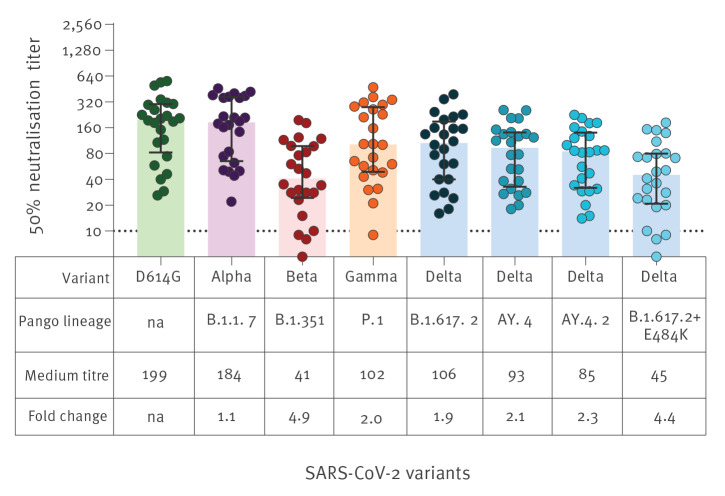
SARS-CoV-2 neutralising antibody titres against variants of concern Alpha, Beta, Gamma and Delta lineage/sub-lineages 2 months after the second dose of Comirnaty (BNT162b2 mRNA) vaccine, Denmark, 1–26 November 2021 (n = 24)

Virus neutralisation titres for other VOC, Alpha (Pango lineage designation B.1.1.7), Beta (Pango lineage designation B.1.351) and Gamma (Pango lineage designation P.1) were available for 24 of the 30 serum samples (Figure 3). Relative to the early pandemic strain (D614G), the reduction in the AY.4.2 sub-lineage-associated virus neutralisation (2.3-fold) was not as pronounced as observed for the Beta variant (4.9-fold). In contrast, the Delta lineage B.1.617.2 with the E484K neutralisation-resistant mutation (4.4-fold) approached the reduction in neutralisation titres observed for the Beta variant.

na: not applicable; Pango: Phylogenetic Assignment of Named Global Outbreak Lineages (Pangolin) designation; SARS-CoV-2: severe acute respiratory syndrome coronavirus 2.

Neutralisation titres for variants of concern Alpha (Pango lineage designation B.1.1.7), Beta (Pango lineage designation B.1.351) and Gamma (Pango lineage designation P.1) in addition to the Delta (Pango lineage designation B.1.617.2) variant strains were available for 24 of the 30 sera from Comirnaty-vaccinated donors. Neutralisation titres were not determined relative to the World Health Organization (WHO) International Standard for anti-SARS-CoV-2 immunoglobulin, human (NIBSC code: 20/136), due to unavailability of the reagent at the time of the study. Solid coloured bars represent the median titre and error bars are the interquartile range. Fold changes are relative to the early pandemic strain D614G.

## Prevalence of AY.4.2. and B.1.617.2 with E484K in Denmark

In Denmark, the first AY.4.2 case was observed on 4 August 2021. In the beginning of September 2021, AY4.2 appeared in more than 2% of sequenced samples but declined to below 1% by mid-September 2021. However, from the last week of October 2021, the percentage of AY.4.2 increased again from 0.5% to 4% by 21 November 2021. In addition to Beta and Gamma, the E484K mutation has emerged in several different lineages, including Delta [8,15]. Between 29 August and 15 November 2021, 56 cases of Delta strains with the E484K mutation have been identified in Denmark. In the UK, 152 sequences of the Delta variant with E484K occurred by 8 November 2021 [2], an increase of 59 cases since an earlier report on 15 October 2021 [7].

### Ethical statement

The study was conducted according to the Declaration of Helsinki. Biological samples were stored as part of the Statens Serum Institut’s diagnostic testing and surveillance. Ethical approval was not required for this surveillance study.

## Discussion

On 20 October 2021, the UK Health Security Agency designated AY.4.2 a Variant Under Investigation (VUI-21OCT-01) on account of a higher growth rate of 19% in the population relative to other Delta lineages and sub-lineages [1]. With additional spike mutations compared to other Delta strains, reduced sensitivity to vaccine-induced antibodies may have contributed to the increase spread. Using a live virus neutralisation assay, we demonstrate that the Delta sub-lineage AY.4.2 virus has a modest reduction of 2.3-fold relative to an early pandemic strain, which is highly homologous to the current vaccine strain. AY.4.2 therefore remains sensitive to vaccine-induced virus neutralisation. Moreover, neutralisation titres for AY.4.2 were not significantly different from the parental B.1.617.2 or AY.4 lineages. It is thus unlikely that neutralisation resistance is a determinant of the increased spread observed for AY.4.2 relative to other Delta lineages in European countries. These findings are in agreement with similar preliminary vaccine effectiveness observed for AY.4.2 compared with non-AY.4.2 Delta cases, both symptomatic and asymptomatic, for the Vaxzevria (ChAdOx1 nCoV-19, Oxford-Astra Zeneca, Cambridge, UK), Comirnaty, and Spikevax (mRNA-1273, Moderna, Cambridge, Massachusetts, US) vaccines in the UK [7]. On the contrary, we show a significant neutralisation resistance for a Delta variant that acquired the E484K spike mutation. This amino acid substitution in the receptor-binding domain, occurring in other VOC such as Beta and Gamma, is associated with reduced sensitivity to monoclonal antibodies, convalescent antisera from early pandemic wave infections, and vaccine elicited anti-sera [6,16,17]. While Delta variants bearing the E484K mutation occur mostly sporadically [8], it has now occurred in Delta sub-lineages with sustained clusters according to recent reports from the UK [7]. 

## Conclusions

The presented data provide further support for continuous monitoring of E484K within emerging Delta sub-lineages, such as the Delta strain examined here. Additional studies of a larger sample size, which evaluate the antigenicity of VOC and circulating Delta variant strains against sera from children and adults vaccinated with different SARS-CoV-2 vaccines modalities (mRNA, recombinant adenovirus, subunit, inactivated) are warranted. These further include evaluations following booster vaccine doses after 4 to 6 months and following natural infection. In addition, similar studies are urgently needed for the recently identified Omicron VOC (Pango lineage designation B.1.1.529) and its sub-lineages [18], which bear different spike mutations.
